# Energy crops affecting farmland birds in Central Europe: insights from a miscanthus-dominated landscape

**DOI:** 10.2478/s11756-018-0143-1

**Published:** 2018-11-05

**Authors:** Jan M. Kaczmarek, Tadeusz Mizera, Piotr Tryjanowski

**Affiliations:** Institute of Zoology, Poznań University of Life Sciences, Wojska Polskiego 71C, 60-625 Poznań, Poland

**Keywords:** Bioenergy, Biomass, Land-use change, Microhabitats, *Miscanthus x giganteus*

## Abstract

The expansion of energy crops such as miscanthus *Miscanthus x giganteus* has changed the habitat of European farmland birds. However, most studies on the subject are based in Western Europe. We surveyed the avian community in a sample bioenergy landscape in Poland to investigate the pattern of use of the crop by birds in Central Europe. During a year-long survey, 80 species were noted, with 32 sighted in miscanthus. The number of bird sightings was negatively correlated with the area covered by miscanthus in study plots. In contrast to results from Western Europe, most woodland-generalist bird species tended to avoid miscanthus. Farmland species (e.g. the corn bunting *Miliaria calandra*; the tree sparrow *Passer montanus*; the starling *Sturnus vulgaris*; the yellowhammer *Emberiza citrinella*) were less frequently sighted in the crop than in other habitats. Only a few species were sighted more often in miscanthus than in other habitats, e.g. the marsh warbler *Acrocephalus palustris*; the reed bunting *Emberiza schoeniclus*; the whinchat *Saxicola rubetra*. The potential impact of expanded miscanthus cropping on bird communities in Central and Eastern Europe may diverge from predictions derived from studies based in Western Europe due to differing habitat preferences and bird densities between the regions. For a majority of farmland species, the crop may constitute only a suboptimal, supplementary habitat, with only a few birds potentially adapting to the exploitation of bioenergy plantations.

**Electronic supplementary material:**

The online version of this article (10.2478/s11756-018-0143-1) contains supplementary material, which is available to authorized users.

## Introduction

Lignocellulosic bioenergy plants, or second-generation energy crops, are considered to have the potential to shape the future of both agricultural and sustainable energy systems (Kumar et al. 2016). Lignocellulosic plants cultivated in temperate zones of the northern hemisphere include miscanthus *Miscanthus x giganteus,* short-rotation willow coppice *Salix viminalis*, reed canary grass *Phalaris arundinacea*, switchgrass *Panicum virgatum*, and several other species (Lewandowski et al. 2003; Rowe et al. 2009). Currently, new species and cultivars are being introduced or developed, sometimes with the help of genetic engineering (Badhan and McAllister 2014; Marriott et al. 2016). Most of these plants are perennial, require low levels of agricultural input, and add some degree of habitat heterogeneity to intensively farmed landscapes, and therefore may support farmland biodiversity and ecosystem services (Rowe et al. 2009; Manning et al. 2015; Holland et al. 2015; Haughton et al. 2009). However, their further expansion may lead to global changes in land use, leading to biodiversity losses (Miyake et al. 2012), and their overall effect on animal and plant communities may be highly complex, including both favourable and adverse aspects (Dauber et al. 2010; Fletcher et al. 2011; Pedroli et al. 2013; Immerzeel et al. 2014).

The expansion of lignocellulosic energy crops is now typically taken into consideration when modelling the future of European farmland bird communities (e.g. Engel et al. 2012; Everaars et al. 2014; Rivas-Casado et al. 2014). However, the body of basic observational research on the use of bioenergy fields by birds remains limited. Additionally, existing research is geographically biased in favour of Western Europe, whereas considerable potential for the development of some lignocellulosic plants exists in Central and Eastern Europe (Castillo et al. 2015). The latter region also holds large areas of high nature value farmland (Tryjanowski et al. 2011; Sutcliffe et al. 2015), which may be threatened by the expansion of energy cropping.

At present, the potentially most important lignocellulosic crop in future European farming landscapes is miscanthus *Miscanthus x giganteus* (Hastings et al. 2009), a fast-growing perennial grass capable of reaching a height of three metres in a single season (Lewandowski et al. 2003). Established miscanthus fields are characterised by a dense structure of tall stems, very different from traditional crops, but providing shelter for birds, including woodland species (Bellamy et al. 2009; Sage et al. 2010), and mammals (Petrovan et al. 2017). Miscanthus cultivars used for bioenergy production are sterile, so the plant itself does not provide any food for granivorous animals (Anderson et al. 2004); however, miscanthus fields can host a variety of weeds, especially during the establishment phase (Semere and Slater 2007). Miscanthus fields are harvested in late autumn, winter, or early spring (Lewandowski et al. 2000); theoretically, therefore, harvesting does not pose a threat to nesting birds (Anderson et al. 2004). However, the crop’s very rapid growth during the breeding season may act as an ecological trap, causing nest abandonment by open-habitat specialists, such as the skylark *Alauda arvensis* L., 1758, or the lapwing *Vanellus vanellus* (L., 1758) (Sage et al. 2010; Bright et al. 2013). Existing studies on bird communities in miscanthus suggest that the expansion of the crop is not necessarily detrimental to farmland bird communities, as it provides additional habitat heterogeneity, an increased supply of weed seed, and a nesting habitat for a variety of farmland and woodland birds (Sage et al. 2010; Bright et al. 2013). However, the relative attractiveness of miscanthus for birds may be an artefact caused by the predominance of fields in the plant’s early establishment phase (Semere and Slater 2007; Bellamy et al. 2009; Bright et al. 2013).

To the best of our knowledge, all field studies investigating bird communities in miscanthus fields have been based in the British Isles, and none of them has included a full year-long survey (Semere and Slater 2007; Bellamy et al. 2009; Sage et al. 2010; Bright et al. 2013; Pringle et al. 2013). As a consequence, some potential effects of the agricultural cycle of miscanthus on birds, like harvesting in late winter/early spring when some species establish their territories, or replacing ploughed fields with tall stands during autumn bird passage, might have gone undetected. Furthermore, the potential large-scale expansion of miscanthus cropping into Central and Eastern Europe (Borzęcka-Walker et al. 2012; Hager et al. 2014; Castillo et al. 2015) requires additional data on avian communities in miscanthus fields. Due to a long-lasting divergence in agricultural practices and land use (e.g. a larger extent of deforestation in Western Europe in the past), habitat preferences of many farmland birds differ between the two regions (Tryjanowski et al. 2011), thus the direct implementation of conservation solutions derived from Western European studies may be inadvisable (Báldi and Batáry 2011). Furthermore, many species are more abundant in Central and East European farmland than in Western Europe (Reif et al. 2008; Vallecillo et al. 2016). Some of these birds may use miscanthus fields as their habitat, a phenomenon which may have remained undetected in Western Europe due to their overall low densities in that region (Kaczmarek and Tryjanowski 2016).

The aim of our study was to fill the gap in knowledge about the use of miscanthus fields by birds outside Western Europe. Central and Eastern European countries differ markedly in their past and present agricultural practices (e.g. very large farm sizes in Czech Republic or Slovakia versus small sizes in Poland, Hungary or Romania). Western Poland, where the study was conducted, couples intensive farming regime (large farm holdings, simplified habitat structure, mechanization) with large and diverse farmland bird populations. Thus, to some extent it may reflect the general patterns found in Central and Eastern Europe, despite a large heterogeneity of the region.

A farmland bird community in a sample bioenergy landscape in Western Poland was investigated in a year-long bird survey, and the results were used to establish whether miscanthus as a dominant crop affects bird abundance and diversity, taking into account the differences between well- and poorly-established miscanthus fields. To identify the species most often detected in miscanthus, we attempted to monitor the seasonal changes in the use of the crop by birds.

## Materials and methods

### Study area

**Fig. 1:**
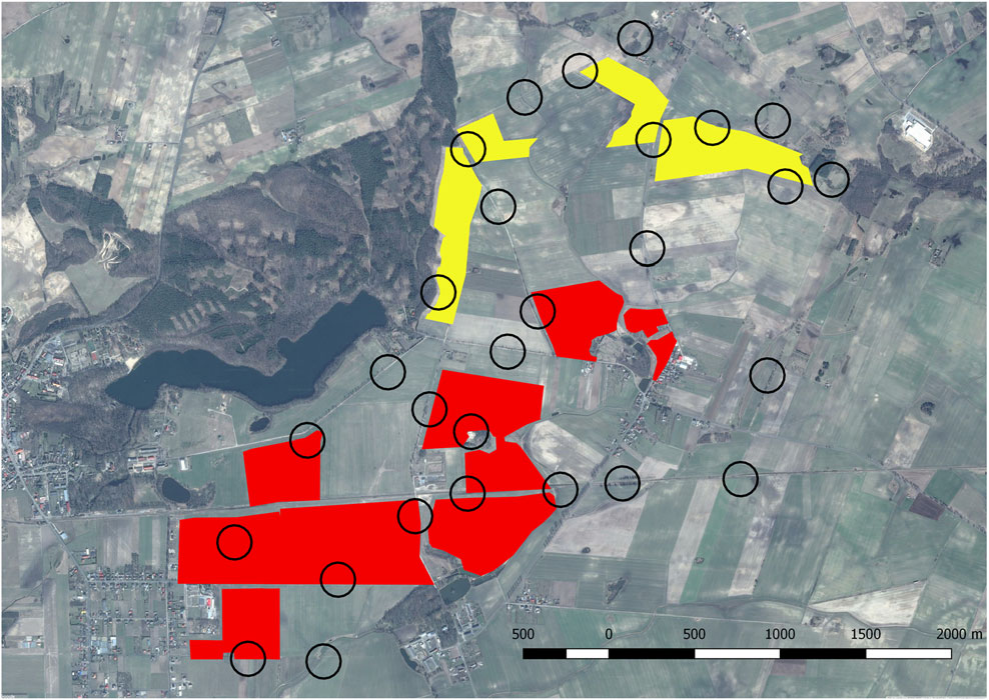
Investigated landscape in W Poland. Circles – study plots. Red polygons – well-established miscanthus fields. Yellow polygons – poorly-established miscanthus fields

The study was conducted between March 2015 and February 2016 in a patch (approximately 1600 ha) of intensive farming landscape in W Poland (N: 52.853; E: 16.566), encompassing 152 ha of well-established miscanthus fields (dense crop covering >80% of the field area) and 89 ha of poorly-established miscanthus fields (patchy crop covering <50% of the field area) (Fig. 1). Miscanthus was harvested between 20th April and 10th May. The crop gained its maximum height in late summer (July–early August). Other crops cultivated in the area were predominantly winter cereals and winter oilseed rape, with small areas of maize and sugar beets. Non-crop vegetation cover consisted of meadows, low marginal vegetation (mostly on field, road, and railway margins), shrubs, orchards, and trees (alley trees and woodland margins). The average field size of miscanthus was 17.59 ± 15.01 ha (mean ± SD), while the average field size of other crops was 8.52 ± 6.13 ha (mean ± SD).

### Bird survey and habitat cover measurements

Data were collected in 28 plots dispersed within the landscape (Fig. 1). Each plot consisted of an observation point and a circular buffer with a radius of 150 m. The buffer width was based on preliminary observations in which small passerine birds perching on miscanthus stalks were identifiable with standard binoculars at distances less than 150 m. The plots were chosen based on expert opinion, according to the following rules: a) clear view of a large area; if possible, hills, railway embankments, hunting stands or bales of straw were used. Alternatively, observation points were placed on dirt roads, where observations were taken from a platform installed on a car roof; b) distance of minimum 400 m from the nearest other observation point; c) heterogeneity, i.e. plots were chosen to enclose as many land cover types as possible. The bird survey was performed on a biweekly (spring and summer) or monthly (autumn and winter) basis. During each session, 5-min bird counts (Gregory et al. 2004) were performed in each plot between dawn and 11 a.m. The order of visits in the plots was changed between the sessions to avoid daytime bias. Birds sighted on the ground, perching, or flushed in one of the habitats within the plot were recorded. Birds flying over the plot from the outside were not recorded; however, an exception was made for the skylark, where birds observed in territorial song flights over the plots were recorded and included in the analyses. Bird survey data were divided into 5 periods: early spring (18th March–12th April, hereafter: spring), core breeding season (22th April–17th July, hereafter: breeding season), late summer (31st July–25th September, hereafter: summer), autumn (11th October–22nd November), and winter (11th December–25th February). During the first visit in each observation plot, all habitats present within the 150-m buffer were classified by the observer into 10 categories (cereals, miscanthus, meadows, other crops, stubble or ploughed fields, low marginal vegetation, shrubs, trees, orchards, open water). Ten plots included various areas of well-established miscanthus, 6 plots included various areas of poorly-established miscanthus, and 12 plots contained no miscanthus (Fig. 1). Any changes in habitat cover (e.g. due to ploughing) were recorded during each visit. After each visit in each plot, the habitat cover in each 150-m buffer was mapped in QGIS 2.10 and all cover areas were measured. To account for changes due to agricultural activity, cover area values for all visits in all plots were totalled for each study period, providing an estimate of relative habitat cover in the plots for each study period as well as throughout the year (Online Resource 1).

### Statistical analyses

The Spearman correlation coefficient was used to reveal potential relationships between miscanthus cover area in plots and number of bird sightings (excluding skylarks) as well as bird diversity (species richness, Shannon-Wiener index of diversity). To assess the effect of the phase of establishment of miscanthus, Kruskal-Wallis ANOVA was used to compare the number of bird sightings (excluding skylarks) per visit in plots where miscanthus was well-established, poorly-established, or absent. Data on the number of skylarks were analysed separately due to a variant survey methodology (i.e. the inclusion of birds in territorial song flight). All the aforementioned analyses were performed on the year-long dataset as well as separately for each season.

For the 10 species most often recorded in miscanthus, bird density proxies (number of sightings/visit/cover area) were calculated for each study period for 4 cover types (miscanthus, cereals, meadows, low marginal vegetation, i.e. the most abundant cover types during the breeding season). However, heterogeneity of standard errors due to low sample sizes prevented us from further testing, and the values are presented for visual inspection only. For these 10 species, only the effect of the miscanthus establishment phase on the number of bird sightings during the whole year was tested (Kruskal-Wallis ANOVA). All analyses were performed in SPSS Statistics 21 (IBM).

## Results

In total, 6568 individuals from 80 avian species were noted in the study area, of which 819 birds from 32 species were sighted in miscanthus fields. The 10 species most often sighted in miscanthus are listed in Table 1; a full list is given in Online Resource 2.

**Table 1:** Bird species most often sighted in miscanthus fields within the studied plots. Number of sightings is given separately for miscanthus and for all other habitats combined (cereals, meadows, low marginal vegetation, other crops, stubble/ploughed fields, shrubs, trees, orchards), summed for all 12 months of the study. Species are ranked according to the number of sightings in miscanthus. The mean cover of miscanthus in the investigated plots throughout the year was 28.94%

Species	N sightings in miscanthus	N sightings in other habitats
Skylark	299	1019
*Alauda arvensis*		
Corn bunting	97	412
*Millaria calandra*		
Yellowhammer	70	497
*Emberiza citrinella*		
Tree sparrow	60	683
*Passer montanus*		
Whinchat	38	27
*Saxicola rubetra*		
Reed bunting	36	21
*Emberiza schoeniclus*		
Blue tit	33	81
*Cyanistes caeruleus*		
Marsh warbler	28	11
*Acrocephalus palustris*		
Starling	22	1019
*Sturnus vulgaris*		
Great tit	19	141
*Parus major*		

In the year-long dataset, there was a marginally significant negative correlation between miscanthus cover in the plots and the number of bird sightings (excluding skylarks). However, the correlation proved non-significant after a sequential Bonferroni correction. When each season was analysed separately, there were significant negative correlations between miscanthus cover in observation plots and the number of bird sightings, as well as species richness, in late summer only (Table 2). In the case of skylarks, there was a significant negative correlation between miscanthus cover and number of sightings in autumn, but it proved non-significant after the sequential Bonferroni correction (Table 3).

**Table 2:** Spearman correlations between overall number of birds sighted (skylarks excluded), species richness, species diversity (Shannon-Wiener diversity index) and area covered by miscanthus in the study plots. Values are given separately for the entire year and for each season. Variation in sample sizes for species diversity is a consequence of a lack of birds, or presence of only a single species, in some plots during at least one visit

Season	Spearman *rho*	*p*	N
Entire year			
N sightings	−0.378	0.048*	28
Species richness	−0.190	0.333	28
Species diversity	0.086	0.664	28
Early spring			
N sightings	−0.297	0.125	28
Species richness	−0.044	0.825	28
Species diversity	−0.130	0.517	27
Breeding season			
N sightings	−0.018	0.927	28
Species richness	0.131	0.505	28
Species diversity	0.011	0.957	28
Late summer			
N sightings	−0.587	0.001**	28
Species richness	−0.456	0.015**	28
Species diversity	0.160	0.415	28
Autumn			
N sightings	−0.279	0.151	28
Species richness	−0.107	0.588	28
Species diversity	−0.068	0.732	28
Winter			
N sightings	0.065	0.744	28
Species richness	0.008	0.967	28
Species diversity	−0.062	0.775	24

*- significant at *p* < 0.05 before sequential Bonferroni correction for multiple testing applied to the whole table

**- significant at *p* < 0.05 after sequential Bonferroni correction for multiple testing applied to the whole table

**Table 3:** Spearman correlations between number of Skylark sightings and area covered by miscanthus in the study plots. Values are given separately for the entire year and for each season. Values for winter were not calculated due to the very low number of birds observed

Season	Spearman *rho*	*p*	N
Entire year	−0.112	0.571	28
Early spring	−0.071	0.718	28
Breeding season	−0.094	0.635	28
Late summer	−0.191	0.329	28
Autumn	−0.427	0.023*	28

*- significant at *p* < 0.05 before the sequential Bonferroni correction applied to the whole table

The number of bird sightings did not differ significantly between plots where miscanthus was well established, poorly established, or absent (K-W ANOVA; Table 4**)**, except for late summer, when plots with well-established miscanthus held significantly fewer birds than plots without miscanthus (Dunn-Bonferroni post hoc test_well established vs. absent_; Z = 9.950; *p* = 0.014).

**Table 4:** Values of Kruskal-Wallis ANOVA of overall number of bird sightings (excluding skylarks) in plots that included well-established miscanthus fields (N_plots_ = 10), poorly-established miscanthus fields (N_plots_ = 6) and no miscanthus fields (N_plots_ = 12)

Season	Bird sightings per plot (mean ± SD)			K-W ANOVA	*p*
	Well-established Miscanthus	Poorly-established Miscanthus	Miscanthus absent		
Whole year	159.8 (±87.37)	209.5 (±184.07)	238.5 (±152.01)	2.048	0.359
Early spring	11.70 (±6.62)	9.17 (±11.58)	33.08 (±43.10)	3.899	0.142
Breeding season	39.60 (±15.48)	38.33 (±36.48)	62.25 (±67.36)	1.222	0.543
Late summer	32.30 (±19.57)	47.00 (±39.46)	95.58 (±66.80)	8.790	0.012*
Autumn	28.30 (±40.50)	100.83 (±151.69)	36.17 (±49.28)	0.429	0.807
Winter	59.88 (±77.64)	14.17 (±22.21)	15.22 (±12.20)	2.610	0.271

*- significant at *p* < 0.05. Values of the Dunn-Bonferroni post hoc tests are given in the text

**Fig. 2:**
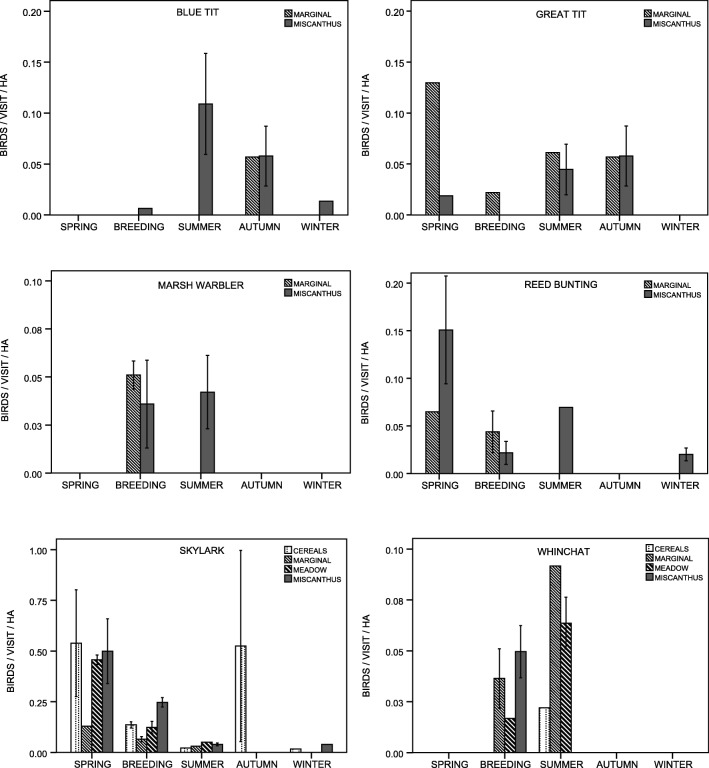
Habitat use of six bird species that reached similar or higher density proxies in miscanthus compared to other key cover types, i.e. cereals, low marginal vegetation, and meadows, during at least one period. Bars represent bird density proxies, i.e. mean (±SE) number of birds sighted per visit per cover area in a given period

**Fig. 3:**
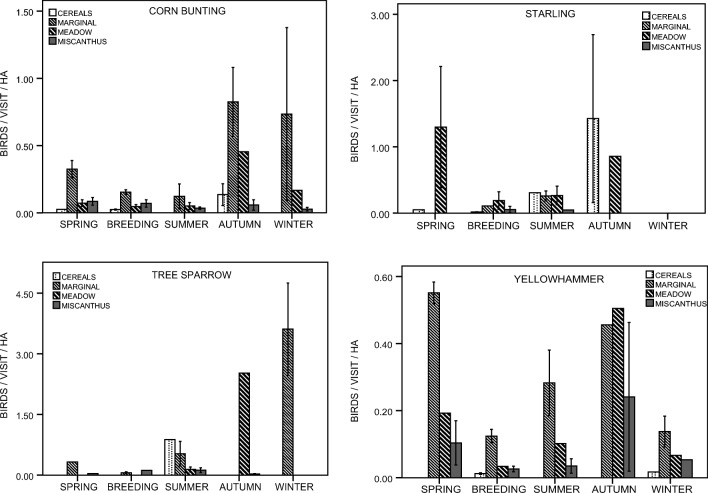
Habitat use of four bird species that consistently reached lower density proxies in miscanthus than in other key cover types, i.e. cereals, low marginal vegetation, and meadows. Bars represent bird density proxies, i.e. mean (±SE) number of birds sighted per visit per cover area in a given period

Among the ten bird species most often noted in miscanthus, six species [blue tit *Cyanistes caeruleus* (L., 1758), great tit *Parus major* (L., 1758), marsh warbler *Acrocephalus palustris* (Bechst., 1798), reed bunting *Emberiza schoeniclus* L., 1758, skylark, whinchat *Saxicola rubetra* (L., 1758)] exhibited similar or marginally higher density proxies in miscanthus than in other key cover types (i.e. cereals, low marginal vegetation, meadows) during at least one period (Fig. 2). In contrast, four species: corn bunting *Millaria calandra* (L., 1758), tree sparrow *Passer montanus* (L., 1758), starling *Sturnus vulgaris* L., 1758, and yellowhammer *Emberiza citrinella* L., 1758, consistently exhibited lower density proxies in miscanthus than elsewhere (Fig. 3).

In three species, there were significant differences in number of bird sightings in plots where miscanthus fields were well-established, poorly-established or absent (K-W ANOVA; Table 5). Marsh warblers were sighted significantly more often in plots with well-established miscanthus than where the crop was absent (Dunn-Bonferroni post hoc test_well-established vs. absent_; Z = −3.646; *p* < 0.001), while reed buntings were more often observed in plots that contained miscanthus, regardless of its establishment phase, than in plots without miscanthus (Dunn-Bonferroni post hoc test_poorly-established vs. absent_; Z = −2.965; *p* = 0.003; Dunn-Bonferroni post hoc test_well-established vs. absent_; Z = −3.532; *p* < 0.001). Starlings were observed significantly more often in plots without miscanthus than in plots with well-established miscanthus (Dunn-Bonferroni post hoc test_well-established vs. absent_; Z = 2.785; *p* = 0.016).

**Table 5:** Kruskal-Wallis ANOVA for overall number of sighting of birds from 10 species observed throughout the year in plots that included well-established miscanthus fields (N_plots_ = 10), poorly-established miscanthus fields (N_plots_ = 6), or no miscanthus fields (N_plots_ = 12)

Species	Bird sightings per plot (mean ± SD)			K-W ANOVA	*p*
	Well-established miscanthus	Poorly-established miscanthus	Miscanthus absent		
Blue tit	4.30 (±3.71)	4.50 (±3.83)	3.67 (±3.58)	0.449	0.799
Corn bunting	14.20 (±11.35)	31.17 (±37.52)	15.00 (±14.98)	1.524	0.467
Great tit	6.90 (±3.57)	2.50 (±4.68)	6.17 ± 5.17	3.574	0.167
Marsh warbler	2.60 (±2.22)	0.83 (±0.98)	0.67 (±2.31)	13.329	0.001*
Reed bunting	3.30 (±2.45)	3.67 (±3.78)	0.17 (±0.39)	15.454	<0.001*
Skylark	18.60 (±6.33)	47.50 (±33.31)	31.58 (±29.62)	2.776	0.250
Starling	3.40 (±6.24)	37.67 (±47.93)	65.08 (±117.41)	7.770	0.021*
Tree sparrow	35.60 (±80.61)	28.83 (±25.60)	17.83 (±32.72)	2.203	0.332
Whinchat	3.20 (±1.93)	2.50 (±2.50)	1.50 (±1.31)	4.886	0.087
Yellowhammer	20.90 (±9.05)	14.00 (±14.10)	22.92 (±18.97)	1.747	0.417

*- significant at *p* < 0.05. Values of the Dunn-Bonferroni post hoc tests are given in the text

## Discussion

Throughout the year, slightly fewer birds were sighted in plots with greater miscanthus cover, although bird species richness and diversity was not significantly affected. When data were analysed separately for each study period, in summer both the number of bird observations and species richness were negatively correlated with miscanthus cover in the study plots.

Due to technical limitations, we were unable to use the methodology of deliberately flushing birds from the tall crop (cf. Sage et al. 2010; Bright et al. 2013). Therefore, reduced bird detectability was to be expected once the crop had reached its maximum height in summer, leading to negative correlations between miscanthus cover and bird abundance. However, the same mechanism should also operate later in the year, whereas no such negative correlations were found for autumn, winter, and early spring. Therefore, although we are aware that the reduced probability of detection in fully-grown miscanthus probably modified our survey results [e.g. relatively few sightings of terrestrial birds such as partridges *Perdix perdix* (L., 1758) or quails *Coturnix coturnix* (L., 1758)], we argue that the observed patterns are a consequence of habitat selection rather than simple detection bias.

The avian community exploiting miscanthus fields consisted almost exclusively of farmland species. The only two woodland/generalist species spotted relatively often in miscanthus were two species of tits, while other woodland species were almost entirely absent. This is in contrast to results from Western Europe, where woodland species such as blackbirds *Turdus merula* dominate avian assemblages in miscanthus in winter (Bellamy et al. 2009) and are abundant in summer. Other species abundant in miscanthus fields in Western Europe in summer include wrens *Troglodytes troglodytes* (L., 1758), chaffinches *Fringilla coelebs* L., 1758, and robins *Erithacus rubecula* (L., 1758) (Sage et al. 2010; Bright et al. 2013). These species were never recorded in miscanthus in our study, despite being present in the landscape, sometimes in large numbers (e.g. chaffinch; see Online resource 2). As forested habitats were present in the landscape and often bordered with the miscanthus fields, local habitat availability was unlikely to be a limiting factor for the woodland birds, and we prefer to interpret the results in the context of habitat selectivity. The habitat preferences of many woodland species differ between Western and Eastern Europe, with birds in the east being more confined to natural woodland habitats (Fuller et al. 2007). For Western Europe, it was suggested that the loss of farmland bird habitats caused by miscanthus expansion might be partly counterbalanced by providing new habitats for woodland species, leaving the overall density and diversity of birds relatively unchanged (Sage et al. 2010). In contrast, in Central and Eastern Europe, the scarcity of woodland species able to exploit miscanthus fields could lead to more apparent adverse effects of miscanthus expansion on bird communities.

Farmland species, such as corn buntings, starlings, tree sparrows, and yellowhammers, reached much lower density proxies in miscanthus than in neighbouring habitats despite their overall abundance in the landscape. Large flocks of birds tend to avoid miscanthus fields outside breeding season (Sage et al. 2010), which was presumably true for starlings and tree sparrows, as these species reached high density proxies in habitats outside miscanthus in early spring and/or autumn. Some other flocking species were almost never observed in miscanthus (e.g. fieldfares *Turdus pilaris* L., 1758, and wood pigeons *Columba palumbus* L., 1758) despite their high numbers in the landscape during passage. The remaining two common farmland species, i.e. corn bunting and yellowhammer, consistently exhibited lower density proxies in miscanthus than in marginal vegetation or meadows, but higher than in cereals. This suggests that miscanthus itself is unlikely to serve as a preferred habitat. Nevertheless, both species were regularly observed in the crop, using dry stalks as song posts or foraging on bare patches within poorly-established miscanthus. The corn bunting is considered potentially threatened by miscanthus expansion in the British Isles (Bright et al. 2013), as it was totally absent or very rare in miscanthus according to all published studies (Semere and Slater 2007; Bellamy et al. 2009; Sage et al. 2010; Bright et al. 2013). In Poland, the population of the species is large and increasing (Krajewska and Mizera 2010; Chief Inspectorate for Environmental Protection 2017), as it probably benefits from moderate agricultural intensification (Szymkowiak et al. 2014). As a consequence, birds from a species abundant in the landscape may keep dispersing into low-quality habitats ‘blurring’ the actual habitat preferences [e.g. red-backed shrike *Lanius collurio* L., 1758 in Tryjanowski et al. 2007].

In contrast to birds abundant in the landscape and relatively seldom recorded in the novel crop, several avian species (whinchat, reed bunting, marsh warbler) were observed predominantly in miscanthus fields. However, none of these species was recorded in miscanthus fields only, which is in agreement with Bellamy et al. (2009), who suggested that miscanthus hosts birds already present in the landscape without attracting any new species. The expansion of miscanthus cropping may provide these birds with an additional habitat. However, the pattern of use of miscanthus fields varies among species, suggesting that the potential harnessing of the novel crop for bird conservation requires a differentiated approach.

For instance, whinchats appeared in miscanthus fields just before and after the harvest, perching on dry miscanthus stalks. We observed both territorial pairs and fledging birds in miscanthus during the breeding season; however, due to the point-count methodology chosen, we did not attempt to locate the nests. As the season progressed and miscanthus grew rapidly, the birds switched to neighbouring habitats, mostly meadows and marginal vegetation. Therefore, miscanthus fields may attract this species during a relatively short period of time, providing song posts and shelter, but losing value for birds afterwards. The use of miscanthus by whinchats was not reported from Western Europe, probably because the species had already withdrawn from intensive farmland in the region, remaining mainly in patches of well-preserved grasslands (Britschgi et al. 2006; Border et al. 2017). In Central and Eastern Europe, the whinchat is still widespread, though declining (Tryjanowski et al. 2009).

Reed buntings were present in miscanthus almost year-round, as the species regularly winters in Western Poland (Tomiałojć and Stawarczyk 2003). In early spring, birds were observed in almost all surveyed plots with miscanthus, but after the harvest (April–May), their numbers were reduced and did not recover. The reed bunting is often reported from miscanthus fields in both summer and winter (Bellamy et al. 2009; Sage et al. 2010; Bright et al. 2013). Our results suggest that miscanthus fields attract numerous reed buntings in the pre-breeding season, when birds roost in optimal habitats before establishing territories (Musilová et al. 2011). Therefore, there is a risk that late harvesting of miscanthus could lead to nesting losses. While reed buntings in Western Europe often nest in crops like oilseed rape (Gruar et al. 2006), in Central and Eastern Europe the species relies mostly on wetlands (Surmacki 2004). Thus, the expansion of miscanthus cropping into the region might facilitate the spread of reed buntings into intensively farmed landscapes.

In contrast to the two previous species, the marsh warbler seems well-suited to the agricultural cycle of miscanthus. In the region, the species returns from its wintering grounds in late May-early June (Tryjanowski et al. 2009), when miscanthus stalks are regaining their maximum height. In our study, marsh warblers were present in almost every miscanthus field surveyed, and were more often sighted in locations with well-established miscanthus. To our knowledge, the marsh warbler has never before been reported from the crop, as it remains an extremely rare breeder in the United Kingdom (Kelsey 1989; Hayhow et al. 2017), where all the previous studies were located. Importantly, the marsh warbler is known to establish territories in dense patches of invasive plants (Skórka et al. 2010; Hajzlerová and Reif 2014). Similarly to the reed bunting, widespread miscanthus cropping might further stimulate expansion of the marsh warbler into intensively farmed landscapes, where it so far relies on wetland and marginal vegetation ‘islands’ for nesting (Surmacki 2005).

The most abundant avian species in the investigated landscape, the skylark, did not clearly avoid miscanthus fields (cf. Bellamy et al. 2009; Sage et al. 2010; Bright et al. 2013). Both in early spring and during the core breeding season, territorial flights were repeatedly observed over miscanthus, along with fledging birds in bare patches in poorly-established miscanthus fields (cf. Bright et al. 2013). However, the phase of miscanthus establishment had no overall effect on skylark abundance. In summer, skylark numbers observed in miscanthus dropped, presumably because of reduced detectability in the mature crop. In autumn, skylarks were never sighted in miscanthus, despite considerable numbers of migrating birds present in the landscape. In spring, the poorly-established miscanthus fields may provide birds with abundant invertebrate food (Bellamy et al. 2009), while in autumn and winter, the stubble of the traditional crops, ploughed or recently sown fields provide easily accessible plant food for skylarks (Donald et al. 2001; Geiger et al. 2014). As a consequence, skylarks on passage may prefer to forage in the open fields instead of miscanthus or meadows.

In conclusion, our results suggest that, on a local scale, the expansion of miscanthus into farming landscapes of Central and Eastern Europe could negatively affect avian communities in a way that differs from predictions based on studies from Western Europe. In contrast to earlier studies, woodland and generalist species were almost absent from miscanthus fields, and most farmland species used the novel crop only to a limited extent. Only a few passerine species were sighted in miscanthus relatively often. However, the results of our year-long survey suggest that even these birds, with the possible exception of the marsh warbler, are likely to strongly depend on the availability of alternative habitats as well as on crop management. Miscanthus remains a marginal crop in Poland, with less than 1000 ha currently planted (The Polish Chamber of Biomass, *pers. comm.*). However, due to economical constraints, it is likely to be planted in large clusters close to biomass-processing facilities, deeply impacting local agricultural landscapes (note that in our study, the average field size of miscanthus was larger than of traditional crops it is likely to replace). As a consequence, while so far the impact of miscanthus cropping on farmland birds probably remains negligible in the country-scale, the novel crop has the potential to affect local bird communities where it is introduced.

## Electronic supplementary material

Online resource 1 (PDF 363 kb)

Online resource 2 (PDF 382 kb)
