# Supplementary material for: Energy crops affecting farmland birds in Central Europe: insights from a miscanthus-dominated landscape
Source: Biologia (Bratisl). 2018 Nov 5;74(1):35–44. doi: 10.2478/s11756-018-0143-1 (PMC6315014; doi:10.2478/s11756-018-0143-1)
Supplement: Supplementary file 1 — (PDF 363 kb) [file 11756_2018_143_MOESM1_ESM.pdf]

Article: Energy crops affecting farmland birds in Central Europe: insights from a miscanthus-dominated landscape

Journal: Biologia

Authors: Jan M. Kaczmarek*, Tadeusz Mizera & Piotr Tryjanowski

*Corresponding author; Institute of Zoology, Poznań University of Life Sciences, Wojska Polskiego 71C, 60-625 Poznań, Poland; email: kaczmarq@up.poznan.pl

Online Resource 1. Land cover in the study plots (i.e. buffers with a 150 m radius around observation points). Values for each visit were summed to provide a standardized estimate of land cover in each season. Differences in values between the seasons are a consequence of varying number of visits to study plots as well as agricultural activity.

	EARLY SPRING		BREEDING SEASON		SUMMER		AUTUMN		WINTER		TOTAL	
COVER TYPE	Area [ha]	%	Area [ha]	%	Area [ha]	%	Area [ha]	%	Area [ha]	%	Area [ha]	%
Cereals	39.89	*23.48*	137.11	*26.89*	45.37	*12.45*	37.12	*18.48*	59.31	*23.41*	318.80	*21.27*
Low marginal vegetation	15.43	*9.08*	45.70	*8.97*	32.73	*8.98*	17.56	*8.75*	21.79	*8.60*	133.21	*8.89*
Meadows	20.79	*12.24*	59.52	*11.68*	39.29	*10.78*	19.80	*9.86*	30.10	*11.88*	169.50	*11.31*
Miscanthus	53.06	*31.23*	153.21	*30.05*	100.93	*27.68*	51.89	*25.83*	74.54	*29.42*	433.63	*28.94*
Orchards	3.36	*1.97*	10.03	*1.97*	6.33	*1.73*	4.82	*2.40*	4.91	*1.94*	29.45	*1.97*
Other crops	9.77	*5.75*	52.70	*10.34*	24.94	*6.84*	17.58	*8.76*	18.60	*7.34*	123.59	*8.25*
Shrubs	1.25	*0.73*	3.70	*0.72*	2.52	*0.69*	1.32	*0.66*	1.59	*0.63*	10.38	*0.69*
Stubble/ploughed fields	17.24	*10.14*	20.29	*3.98*	93.90	*25.76*	39.84	*19.84*	28.61	*11.29*	199.88	*13.34*
Trees	9.16	*5.39*	27.55	*5.40*	18.58	*5.09*	10.91	*5.43*	13.90	*5.49*	80.10	*5.35*
SUM	169.93	*100.00*	509.80	*100.00*	364.59	*100.00*	200.83	*100.00*	253.35	*100.00*	1498.50	*100.00*
