# Supplementary material for: Energy crops affecting farmland birds in Central Europe: insights from a miscanthus-dominated landscape
Source: Biologia (Bratisl). 2018 Nov 5;74(1):35–44. doi: 10.2478/s11756-018-0143-1 (PMC6315014; doi:10.2478/s11756-018-0143-1)
Supplement: Supplementary file 2 — (PDF 382 kb) [file 11756_2018_143_MOESM2_ESM.pdf]

Article: Energy crops affecting farmland birds in Central Europe: insights from a miscanthus-dominated landscape

Journal: Biologia

Authors: Jan M. Kaczmarek*, Tadeusz Mizera & Piotr Tryjanowski

*Corresponding author; Institute of Zoology, Poznań University of Life Sciences, Wojska Polskiego 71C, 60-625 Poznań, Poland; email: kaczmarq@up.poznan.pl

Online Resource 2

Online Resource 2. Species list with a number of sightings of each species during the study. The number of sightings is shown for miscanthus fields and for all other habitats combined.

Species	Miscanthus	Other habitats	Species	Miscanthus	Other habitats
Skylark *Alauda arvensis* L., 1758	299	1019	Grasshopper warbler *Locustella naevia* (Bodd., 1783)	-	1
Marsh warbler *Acrocephalus palustris* (Bechst., 1798)	28	11	Woodlark *Lullula arborea* (L., 1758)	3	1
Reed warbler *Acrocephalus scirpaceus* (Herm., 1804)	-	2	Rufous nightingale *Luscinia megarhynchos* Brehm, 1831	-	7
Linnet *Carduelis cannabina* (L., 1758)	11	36	Corn bunting *Miliaria calandra* (L., 1758)	97	412
Sparrowhawk *Accipitter nisus* (L., 1758)	-	2	Spotted flycatcher *Muscicapa striata* (Pall., 1764)	-	1
Long-tailed tit *Aegithalos caudatus* (L., 1758)	-	12	White wagtail *Motacilla alba* L., 1758	1	17
Mallard *Anas platyrhynchos* L., 1758	2	4	Yellow wagtail *Motacilla flava* L., 1758	16	35
Meadow pipit *Anthus pratensis* (L., 1758)	-	16	Northern wheatear *Oenanthe oenanthe* (L., 1758)	1	5
Grey heron *Ardea cinerea* L., 1758	-	2	Golden oriole *Oriolus oriolus* (L., 1758)	-	14
Buzzard *Buteo buteo* (L., 1758)	2	30	Tree sparrow *Passer montanus* (L., 1758)	60	683
Rough-legged buzzard *Buteo lagopus* (Pont., 1763)	-	1	Pheasant *Phasianus colchicus* L., 1758	-	1
Greenfinch *Carduelis chloris* (L., 1758)	-	34	Chiffchaff *Phylloscopus collybita* (Vieill., 1817)	1	15
Goldfinch *Carduelis carduelis* (L., 1758)	12	158	Wood warbler *Phylloscopus sibilatrix* (Bechst., 1793)	-	1
White stork *Ciconia ciconia* (L., 1758)	-	8	Willow warbler *Phylloscopus trochilus* (L., 1758)	-	4
Marsh harrier *Circus aeruginosus* (L., 1758)	-	2	Magpie *Pica pica* (L., 1758)	-	20
Montagu's harrier *Circus pygargus* (L., 1758)	-	1	Great tit *Parus major* L., 1758	19	141
Stock dove *Columba oenas* L., 1758	-	50	Willow tit *Poecile montanus* (Conrad, 1827)	1	1
Hooded crow *Corvus cornix* L., 1758	-	97	Black redstart *Phoenicurus ochruros* (Gmel., 1774)	-	3
Raven *Corvus corax* L., 1758	15	99	Green woodpecker *Picus viridis* L., 1758	-	6
Woodpigeon *Columba palumbus* L., 1758	-	345	Grey partridge *Perdix perdix* (L., 1758)	9	3
Quail *Coturnix coturnix* (L., 1758)	5	9	Bullfinch *Pyrrhula pyrrhula* (L., 1758)	-	7
Siskin *Carduelis spinus* (L., 1758)	-	118	Goldcrest *Regulus regulus* (L., 1758)	-	3
Hawfinch *Coccothraustes coccothraustes* (L., 1758)	-	9	Starling *Sturnus vulgaris* L., 1758	22	1019
Cuckoo *Cuculus canorus* L., 1758	-	4	Whitethroat *Sylvia communis* Lath., 1787	4	51
Blue tit *Cyanistes caeruleus* (L., 1758)	33	81	Blackcap *Sylvia atricapilla* (L., 1758)	-	18
Great spotted woodpecker *Dendrocopos major* (L., 1758)	-	15	Collared dove *Streptopelia decaocto* (Friv., 1838)	-	2
Lesser spotted woodpecker *Dryobates minor* (L., 1758)	-	1	Nuthatch *Sitta europaea* L., 1758	-	7
Black woodpecker *Dryocopus martius* (L., 1758)	-	13	Stonechat *Saxicola rubicola* (L., 1766)	13	7
Robin *Erithacus rubecula* (L., 1758)	-	6	Whinchat *Saxicola rubetra* (L., 1758)	38	27
Yellowhammer *Emberiza citrinella* L., 1758	70	497	Serin *Serinus serinus* (L., 1766)	-	3
Reed bunting *Emberiza schoeniclus* L., 1758	36	21	Lesser whitethroat *Sylvia curruca* (L., 1758)	-	6
Kestrel *Falco tinnunculus* L., 1758	-	2	Wren *Troglodytes troglodytes* (L., 1758)	-	2
Chaffinch *Fringilla coelebs* L., 1758	1	208	Song thrush *Turdus philomelos* Brehm, 1831	-	12
Jay *Garrulus glandarius* (L., 1758)	2	57	Blackbird *Turdus merula* L., 1758	8	57
Crested lark *Galerida cristata* (L., 1758)	-	2	Mistle thrush *Turdus viscivorus* L., 1758	1	-
Crane *Grus grus* (L., 1758)	3	43	Filedfare *Turdus pilaris* L., 1758	-	465
Barn swallow *Hirundo rustica* L., 1758	-	1	Hoopoe *Upupa epops* L., 1758	-	3
Icterine warbler *Hippolais icterina* (Vieill., 1817)	-	13	Lapwing *Vanellus vanellus* (L., 1758)	-	21
Wryneck *Jynx torquilla* L., 1758	-	5			
